# Targeting RNA N6-methyladenosine to synergize with immune checkpoint therapy

**DOI:** 10.1186/s12943-023-01746-6

**Published:** 2023-02-21

**Authors:** Xianyong Zhou, Chen Li, Tong Chen, Wenhao Li, Xiaolong Wang, Qifeng Yang

**Affiliations:** 1 Department of Breast Surgery, General Surgery, Qilu Hospital, Cheeloo College of Medicine, Shandong University, Wenhua Xi Road No. 107, Jinan, Shandong China; 2 Department of Breast Surgery, Binzhou People’s Hospital, Binzhou, Shandong China; 3 Department of Pathology Tissue Bank, Qilu Hospital, Cheeloo College of Medicine, Shandong University, Xi Road No. 107, Shandong Jinan, China; 4 Research Institute of Breast Cancer, Shandong University, Jinan, Shandong China

**Keywords:** Cancer immunotherapy, Immune checkpoint, Epigenetics, N6-methyladenosine, m^6^A regulators

## Abstract

Cancer immunotherapy, especially immune checkpoint therapy, has revolutionized therapeutic options by reactivating the host immune system. However, the efficacy varies, and only a small portion of patients develop sustained antitumor responses. Hence, illustrating novel strategies that improve the clinical outcome of immune checkpoint therapy is urgently needed. N6-methyladenosine (m^6^A) has been proved to be an efficient and dynamic posttranscriptional modification process. It is involved in numerous RNA processing, such as splicing, trafficking, translation and degradation. Compelling evidence emphasizes the paramount role of m^6^A modification in the regulation of immune response. These findings may provide a foundation for the rational combination of targeting m^6^A modification and immune checkpoints in cancer treatment. In the present review, we summarize the current landscape of m^6^A modification in RNA biology, and highlight the latest findings on the complex mechanisms by which m^6^A modification governs immune checkpoint molecules. Furthermore, given the critical role of m^6^A modification in antitumor immunity, we discuss the clinical significance of targeting m^6^A modification to improve the efficacy of immune checkpoint therapy for cancer control.

## Background

The immune system functions as a guardian of the host by initiating immune responses against harmful pathogens and tumor cells. However, undue response is responsible for chronic or exaggerated inflammation and autoimmune diseases. Immune checkpoints play pivotal roles in regulating the magnitude of immune responses, thus maintaining immune homeostasis and self-tolerance. Whereas, tumor cells could also utilize immune checkpoints to evade immune surveillance, inhibit antitumor immune response, and eventually result in tumorigenesis and tumor progression [1]. Immune checkpoint therapy, a monoclonal antibody-based blockade, has driven a revolution in the field of cancer treatment [2]. To date, a variety of immune checkpoint inhibitors (ICIs) have been approved by the Food and Drug Administration (FDA) for clinical application [3]. However, the low response rate, innate or acquired drug resistance and immunotherapy-related adverse events (irAEs) challenge the utility of clinical application. Therefore, the discovery of novel strategies that can be combined with ICIs is of utmost importance to maximize therapeutic benefit.

RNA modification has been reported for more than 70 years [4]. Due to the advancements in high-throughput sequencing technology, at least 160 types of RNA modifications have been identified [5]. N6-methyladenosine (m^6^A), the methylation of adenosine at the N6 site, is regarded as the foremost and most abundant posttranscriptional process in eukaryotic messenger RNAs (mRNAs) and noncoding RNAs (ncRNAs) [6]. Approximately 1 – 4 m^6^A sites are detected in 1000 adenosine nucleotide residues [7]. As shown in a previous study, m^6^A modification mainly occurs in the “RRACH” sequence (where R = A or G; H = A, C, or U) [7], and is highly enriched in the 3' untranslated region (3' UTR), in the vicinity of stop codons in mRNAs or near the last exon in ncRNAs [7, 8]. Increasing evidence has revealed that m^6^A modification is involved in multiple aspects of biological activities, especially tumorigenesis and tumor progression [9].

The functions of m^6^A modification and immune checkpoints have been studied extensively in recent decades, and a range of immune checkpoints have been reported to be supervised in an m^6^A-dependent manner. In this review, we introduce the biological roles of m^6^A modification and immune checkpoints, and elaborate the recent progress in understanding the molecular mechanisms underlying m^6^A-modified immune checkpoints. To verify the potency of m^6^A in clinical application, we provide an overview of the up-to-date knowledge and future perspectives of m^6^A modification in cancer treatment. Furthermore, recent attempts to combine targeting m^6^A modification and immune checkpoints as a promising synergistic strategy for cancer therapy, as well as the potential limitations, are discussed.

### RNA m^6^A modification and the regulators

**Fig. 1:**
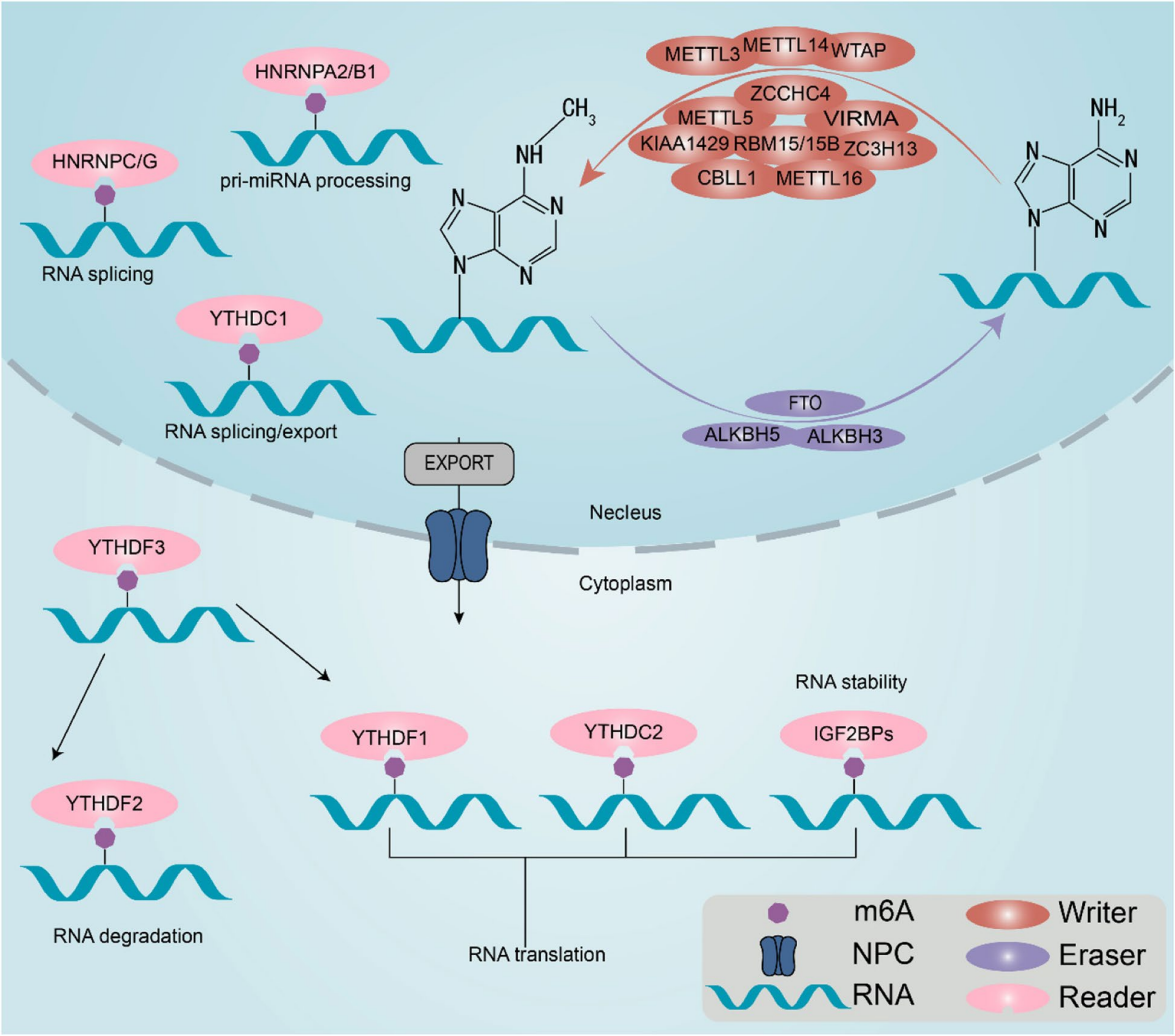
Mechanisms of RNA m^6^A modification. The adenosine (**A**) in mRNA is methylated to form N6-methyladenosine (m^6^A) via the core subunit of the methyltransferase complex (MTC) constitute by METTL3-METTL14-WTAP, and other writers, such as METTL5, METTL16, VIRMA, ZCCHC4, ZC3H13, CBLL1, RBM15 and RBM15B. m^6^A can be removed by erasers, including FTO, ALKBH3 and ALKBH5. The biological roles of m^6^A in RNA metabolism are exerted by readers, including YTHDF1/2/3, YTHDC1/2, IGF2BP1/2/3, and HNRNPC/HNRNPG/HNRNPA2B1

RNA m^6^A modification, a dynamic, reversible and multilayered posttranscriptional process [10], is orchestrated by three types of enzymes: methyltransferases (writers), demethylases (erasers) and binding proteins (readers) [11]. On account of the great breakthrough in epitranscriptomics, an increasing number of writers, erasers and readers have been discovered. In the following section, we summarize the current landscapes of m^6^A regulators in RNA metabolism (Table 1, Fig. 1).

**Table 1:** Functions of m^6^A regulators

Type	Modulator	Function	References
Writer	METTL3	Catalyzes m^6^A methylation	[14]
	METTL14	Stabilizes METTL3 and recognizes target RNAs	[15]
	WTAP	Provides a structural support to stabilize the MTC, and recruits METTL3 and METTL14 to nuclear speckles	[16]
	VIRMA	Locates the MTC near the 3' UTR and stop codon regions of RNAs	[17]
	RBM15/15B	Recruits METTL3 and WTAP to specific RNA sites	[17, 18]
	ZC3H13	Retains the MTC in nuclear speckles by interacting with WTAP and improves the catalytic function of the MTC	[18, 19]
	ZCCHC4	Adds m^6^A to the 28S ribosome rRNAs	[20]
	CBLL1	Stays attached with additional regulators and regulates m^6^A modification	[19]
	METTL5	Participates in 18S rRNA m^6^A modification	[21]
	METTL16	Catalyzes m^6^A installation in the 3' UTR of mRNA and A43 of U6 snRNA	[23, 24]
Eraser	FTO	Removes m^6^A from RNAs	[28]
	ALKBH5	Removes m^6^A from RNAs	[29]
	ALKBH3	Removes m^6^A from tRNAs or acts as an m1A demethylase	[42, 43, 139]
Reader	YTHDF1	Initiates RNA translation	[45]
	YTHDF2	Mediates mRNA degradation	[46]
	YTHDF3	Acts as fortifier to YTHDF1 and YTHDF2	[47]
	YTHDC1	Promotes RNA splicing and exporting, and mediates X-chromosome silencing	[48, 49]
	YTHDC2	Increases the translation capacity and decreases the abundance of target mRNAs	[51]
	IGF2BP1/2/3	Promotes the translation and stability of target mRNAs	[52]
	HNRNPA2B1	Promotes primary miRNAs processing and regulates RNA exporting	[53]
	HNRNPC/G	Modulates the abundance and splicing of target mRNAs	[54]

### ***m***^***6***^***A methyltransferases/writers***

The m^6^A modification is catalyzed via the formation of a methyltransferase complex (MTC) that contains several writers, including methyltransferase-like 3 (METTL3), methyltransferase-like 5 (METTL5), methyltransferase-like 14 (METTL14), methyltransferase-like 16 (METTL16), Wilms tumor 1-associated protein (WTAP), Vir-like m^6^A methyltransferase associated (VIRMA, also called KIAA1429), zinc finger CCHC-type containing 4 (ZCCHC4), zinc finger CCCH-type containing 13 (ZC3H13), Cbl proto-oncogene-like 1 (CBLL1, also known as HAKAI), and RNA-binding motif protein 15 (RBM15) and its paralogue RBM15B [12, 13].

METTL3, METTL14 and WTAP constitute the core subunits of the MTC. As the unique catalytic component of MTC, METTL3 alone carries limited catalytic activity, which is markedly intensified when it is integrated with METTL14 [14]. METTL14 shows no catalytic activity, but is essential for recognizing substrate RNAs, and functions as a scaffold for METTL3 [15]. In the writing process, WTAP stabilizes the core MTC, formed by METTL3 and METTL14, and promotes m^6^A installation by recruiting the complex to nuclear speckles [16]. The other subunits carry specific functions. VIRMA transports the MTC to the 3' UTR or stop codon regions of mRNAs [17]. RBM15 and its paralogue RBM15B facilitate the binding of METTL3 and WTAP, and then recruit the complex to specific RNA-binding sites [17, 18]. By interacting with WTAP, ZC3H13 maintains the MTC in nuclear speckles and increases its catalytic function [19]. ZCCHC4 is critical for adding m^6^A to the 28S ribosomal RNAs (rRNAs), and mediating the distribution and translation of rRNAs [20].

In addition to the canonical writers, many novel writers with diverse functions have been identified in recent studies. METTL5 participates in the m^6^A modification of 18S rRNAs [21]. Similar to the METTL3-METTL14 complex, METTL5 shows metabolic stability by forming a heterodimeric complex with its coactivator TRMT112 [21]. METTL16 is a recently discovered, independent and active writer. It drives m^6^A at the 3' UTR of mRNAs and at the A43 site of U6 small nuclear RNAs (snRNAs) [22]. Additionally, METTL16 plays a crucial role in RNA stability and splicing regulation [23, 24]. Phosphorylated CTD interacting factor 1 (PCIF1), a cap-specific adenosine methyltransferase (CAPAM), mediates m^6^A modification on 2'-O-methylated adenosine present at the 5' end of mRNAs (m^6^Am) [25].

### ***m***^***6***^***A demethylases/erasers***

The erasers, capable of removing m^6^A from RNAs, enable m^6^A modification to be a dynamic and reversible process. To date, two proteins, fat mass- and obesity- associated protein (FTO) and AlkB homolog 5 (ALKBH5), have been identified as canonical m^6^A erasers [26, 27]. As two members of the Fe^2+^/α-ketoglutarate-dependent dioxygenase family, FTO and ALKBH5 can successively convert m^6^A to N6-hydroxymethyladenosine (hm^6^A), N6-formyladenosine (f6A) and adenosine [28]. Recent studies have indicated that ALKBH5 may function in a more direct manner by removing the methyl group of m^6^A, converting m^6^A to adenine [29]. Additionally, FTO can catalyze the demethylase activity on other types of RNA methylation, including N6,2'-O-dimethyladenosine (m^6^Am) on mRNAs and snRNAs, as well as m1A on tRNAs [28, 30]. Overexpression of FTO has been found in a set of tumors, such as glioblastoma (GBM), melanoma, breast cancer (BC) and acute myeloid leukemia (AML), highlighting the m^6^A-dependent oncogenic role of FTO, as well as the tight relationship between impaired FTO expression and short survival time in the cancer context [31–34]. ALKBH5 seems to specifically demethylate m^6^A on single-stranded RNAs [29]. Dysregulated in numerous malignancies, ALKBH5 plays oncogenic or antitumor roles as an m^6^A demethylase, depending on the tumor type. Studies have revealed that ALKBH5 functions as a tumor promotor in GBM, gastric cancer, ovarian cancer and AML [35–38]. However, an antitumor role of ALKBH5 has been demonstrated in hepatocellular carcinoma (HCC), pancreatic cancer and non-small cell lung cancer (NSCLC) [39–41]. In addition, ALKBH3 is defined as a novel and multifunctional m^6^A eraser. In contrast to mRNA or rRNA, tRNA is the preferred substrate of ALKBH3 [42]. Furthermore, ALKBH3 has been reported to function as an m1A demethylase [43].

### ***m***^***6***^***A binding proteins/readers***

The biological function of m^6^A is performed by the recruitment of certain binding proteins, which selectively integrate with methylated RNAs to affect their metabolism. Readers of m^6^A are mainly comprised of YT521-B homology (YTH) domain family proteins (YTHDF1/2/3), YTH domain-containing proteins (YTHDC1/2), insulin-like growth factor 2 mRNA-binding proteins (IGF2BP1/2/3) and heterogeneous nuclear ribonucleoproteins (HNRNPC/HNRNPG/HNRNPA2B1). YTHDF1/2/3 and YTHDC1/2 have been extensively studied and can be classified into three types according to their cellular location: cytoplasmic YTHDF1, YTHDF2 and YTHDF3; nucleocytoplasmic YTHDC2; and nuclear YTHDC1 [44]. YTHDF1 has been reported to bind to m^6^A near the stop codons of mRNAs and recruit factors to initiate RNA translation [45]. YTHDF2 selectively binds to m^6^A-methylated mRNAs and mediates their decay by recruiting the CCR4–NOT deadenylase complex [46]. YTHDF3 functions as a fortifier to YTHDF1 and YTHDF2 [47]. As a nuclear reader, YTHDC1 recruits and modulates serine- and arginine-rich splicing factor 3 (SRSF3) to localize to the binding site of pre-mRNAs, and regulates their alternative splicing [48]. In addition, YTHDC1 recognizes the target mRNAs and ncRNAs to facilitate their exporting from nucleus [49]. Recent studies have revealed that YTHDC1 induces the decay of chromosome-associated regulatory RNAs (carRNAs), including promoter-associated RNAs, enhancer RNAs and repeat RNAs [50]. YTHDC2 increases the translation capacity of target mRNAs, while decreases their abundance [51]. IGF2BP1/2/3 are involved in promoting the translation and stability of mRNAs by recognizing the consensus GG(m^6^A)C sequence [52]. By recognizing primary miRNAs (pri-miRNAs), HNRNPA2B1 promotes the maturation of miRNAs [53]. Both HNRNPC and HNRNPG are known as indirect readers since they do not bind to the m^6^A modification sites directly, but selectively bind to the newly formed structure altered by m^6^A modification, which is called “m^6^A switch” [54].

### Immune checkpoints

**Fig. 2:**
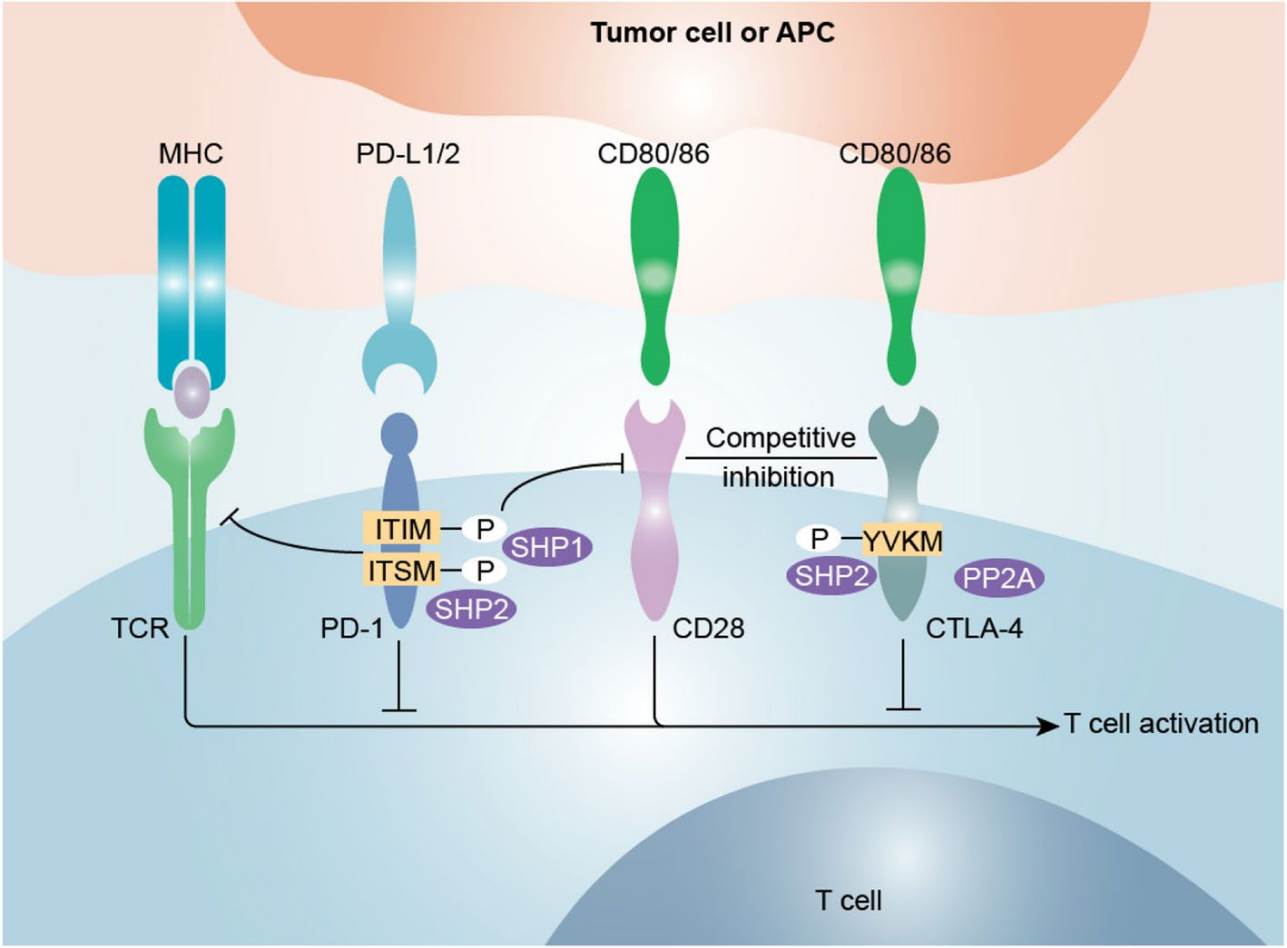
Mechanisms of inhibition of T cell activation induced by CTLA-4 and PD-1. Tumor cells or antigen-presenting cells transmit stimulatory and inhibitory signals to modulate T cell activation. The T cell receptor (TCR) interacts with the major histocompatibility complex (MHC)-peptide complex presented by antigen-presenting cells (APCs). CD28-CD80/CD86 interaction induced costimulatory signaling promotes T cell activation. Cytotoxic T lymphocyte antigen 4 (CTL A-4) decreases CD28 signaling by competing for the shared ligands (CD80/CD86). The CTLA-4-CD80/CD86 interaction induces inhibitory signaling by protein phosphatase 2A (PP2A) or Src homology 2 domain-containing phosphatase 2 (SHP2). Upon the binding of programmed cell death 1 (PD-1) with programmed cell death 1 ligand 1 (PD-L1)/programmed cell death 1 ligand 1 (PD-L2), the immunoreceptor tyrosine-based inhibitory motif (ITIM) and immunoreceptor tyrosine-based switch motif (ITSM) motifs in PD-1 are phosphorylated, resulting in the recruitment of phosphatases SHP1 and SHP2. This activity leads to the dephosphorylation of key molecules in the TCR and CD28 signaling pathways, thereby inhibiting T cell activation

Immune checkpoints, which function as brakes, play essential roles in maintaining homeostasis of the immune system by limiting the magnitude of immune response. Owing to their durable efficacy and minimal side effects, ICIs have revolutionized cancer treatment and led to immunotherapy being adopted as a treatment strategy following surgery, chemotherapy and radiotherapy [55, 56]. A large number of immune checkpoints have been explored in recent decades, including but not limited to cytotoxic T lymphocyte-associated protein-4 (CTLA-4), programmed cell death protein-1 (PD-1), programmed cell death ligand 1 (PD-L1), T cell immunoglobulin and mucin domain-containing-3 (TIM-3), lymphocyte activation gene-3 (LAG-3), T cell immunoglobulin and ITIM domain (TIGIT), V-domain Ig suppressor of T cell activation (VISTA), B7 homolog 3 (B7-H3) and B and T cell lymphocyte attenuator (BTLA) [57]. Our review focuses mainly on the most extensively studied immune checkpoints, namely, CTLA-4, PD-1 and PD-L1, whose expression has been reported to be manipulated in an m^6^A-dependent manner (Table 2, Fig. 2). The interplay between the m^6^A modification and other immune checkpoints is still being elucidated.

**Table 2:** Summary of the biological and molecular functions of CTLA-4, PD-1 and PD-L1

Molecule	Ligand(s)	Receptor expression pattern	Biological function	Molecular mechanism	References
CTLA-4	CD80 (B7-1), CD86 (B7-2)	Activated T cells, Tregs, NK cells	Restrains T cell activity and T-cell co-stimulation	Competitively inhibits CD28 signaling (binding of CD80 and CD86); reduces CD80/CD86 expression	[58, 62, 63]
PD-1	PD-L1, PD-L2	Activated T cells, Tregs, NK cells, NKT cells, B cells, macrophages, DCs, Langerhans’ cells	Inhibits T-cell stimulation and costimulatory signaling	Inhibits TCR signaling and CD28 signaling	[68, 73, 76, 78, 80]
PD-L1	PD-1	DCs, monocytes, macrophages, mast cells, T cells, B cells, NK cells, tumor cells	Suppresses T-cell activity	Inhibits T-cell activity by binding to PD-1	[70–73]

*Tregs* Regulatory T cells, *NK cells* Natural killer cells, *NKT cells* Natural killer T cells, *DCs* Dendritic cells

### Cytotoxic T-lymphocyte associated protein 4 (CTLA-4)

CTLA-4 is predominantly expressed on T cells and to a lesser degree on activated B cells, dendritic cells (DCs), monocytes and granulocytes. CTLA-4 has been identified as a negative regulator of T cell activation after antigenic stimulation of the T-cell receptor (TCR) [58]. The full activation of T cells requires three signaling pathways: (1) TCR binding with the major histocompatibility complex (MHC)–peptide complex presented by antigen-presenting cells (APCs); (2) CD80 (B7-1)/CD86 (B7-2) on APCs integrating with the costimulatory molecule CD28 on T cells; and (3) cytokines produced by T cells regulating immune responses [59].

As a transmembrane protein, CTLA-4 mainly localizes in the intracellular compartments. Upon the activation of T cells, CTLA-4 is translocated to the cell surface. TCR signaling increases CTLA-4 glycosylation and surface level abundance by stimulating hexosamine metabolism and the N-glycan-branching pathway [60]. CTLA-4 in exosomes can be recycled back to the cell surface to increase its surface expression level [61]. CTLA-4 functions as a major coinhibitory receptor by competing with CD28 for the shared B7 ligands (CD80/CD86) during the priming phase of immune response [62]. It decreases CD28 signaling mainly in two ways. First, CTLA-4 on T cells reduces the expression of CD80/CD86 on APCs in a trans-endocytosis manner [63]. Second, CTLA-4 binds to CD80/CD86 with a higher affinity than CD28 and initiates an inhibitory signaling mediated through phosphatases, such as protein phosphatase 2A (PP2A) or Src homology 2 domain-containing phosphatases (SHPs) [64]. PP2A is a vital serine-threonine phosphatase, assisting in maintaining cell homoeostasis by blocking kinase-mediated intracellular signaling pathways [65]. Upon the activation of TCR, CTLA-4 associates with PP2A to suppress the phosphatidylinositol 3-kinase (PI3K)/AKT pathway [66]. In addition, SHP2 can be recruited by the YVKM motif in CTLA-4 cytoplasmic domain to inhibit T cell activation [67].

### PD-1/PD-L1

PD-1 is a negative regulator of immune response. It is abundantly expressed on the surface of most activated immune cells, such as B cells, T cells, macrophages, DCs and Langerhans’ cells [68]. As a type I transmembrane protein receptor, PD-1 contains an immunoglobulin V (Ig-V)-like extracellular domain, a transmembrane domain and a cytoplasmic domain [69]. Two motifs in the cytoplasmic domain of PD-1, named immunoreceptor tyrosine-based inhibitory motif (ITIM) and immunoreceptor tyrosine-based switch motif (ITSM), are essential for the inhibitory function of PD-1.

PD-L1 and PD-L2 are two ligands for PD-1, which belong to the B7 family of type I transmembrane proteins. PD-L1, the production of which is induced by IFN-γ, is primarily expressed on endothelial cells, tumor cells and activated leukocytes, including T cells, natural killer (NK) cells, B cells, DCs and macrophages. PD-L2, the expression of which is regulated by IL-4, is predominantly found on activated DCs and macrophages [70]. Both of the ligands consist of two extracellular domains (an Ig-V domain and Ig-C domain), a transmembrane domain and a short cytoplasmic domain that carries nonclassical signaling motifs with unclear functions [71]. PD-1 antagonists, which block binding of both PD-L1 and PD-L2 to PD-1, show clinical efficiency similar to that of PD-L1 antagonists. Therefore, PD-L1, but not PD-L2, is believed to be the dominant inhibitory ligand of PD-1 [72].

PD-1 exerts its inhibitory effect on T cells primarily through intracellular signaling pathway. Engagement of PD-1 with its ligands results in phosphorylation of ITIM and ITSM motifs [73]. Phosphorylated ITSM and ITIM are critical for the recruitment of phosphatases SHP1 and SHP2, which in turn dephosphorylate key molecule in TCR and CD28 signaling pathways [74, 75], thereby attenuating downstream pathways, such as the PI3K/AKT, ZAP70, RAS, ERK, VAV and phospholipase Cγ (PLCγ) pathways [74, 76–80]. All these pathways ultimately lead to dampened T cell activation, proliferation and cytokine production. Moreover, even with the SHP1/2 genes knocked down, PD-1 can inhibit proliferation and cytokine production in primary T cells, suggesting an alternative signaling mechanism that initiates PD-1 signaling pathway activation [81]. Additionally, PD-L1-containing exosomes secreted by tumor cells can activate the PD-1 pathway and suppress T cell activity in draining lymph nodes [82].

### Role of m^6^A modification in regulating immune checkpoints

**Fig. 3:**
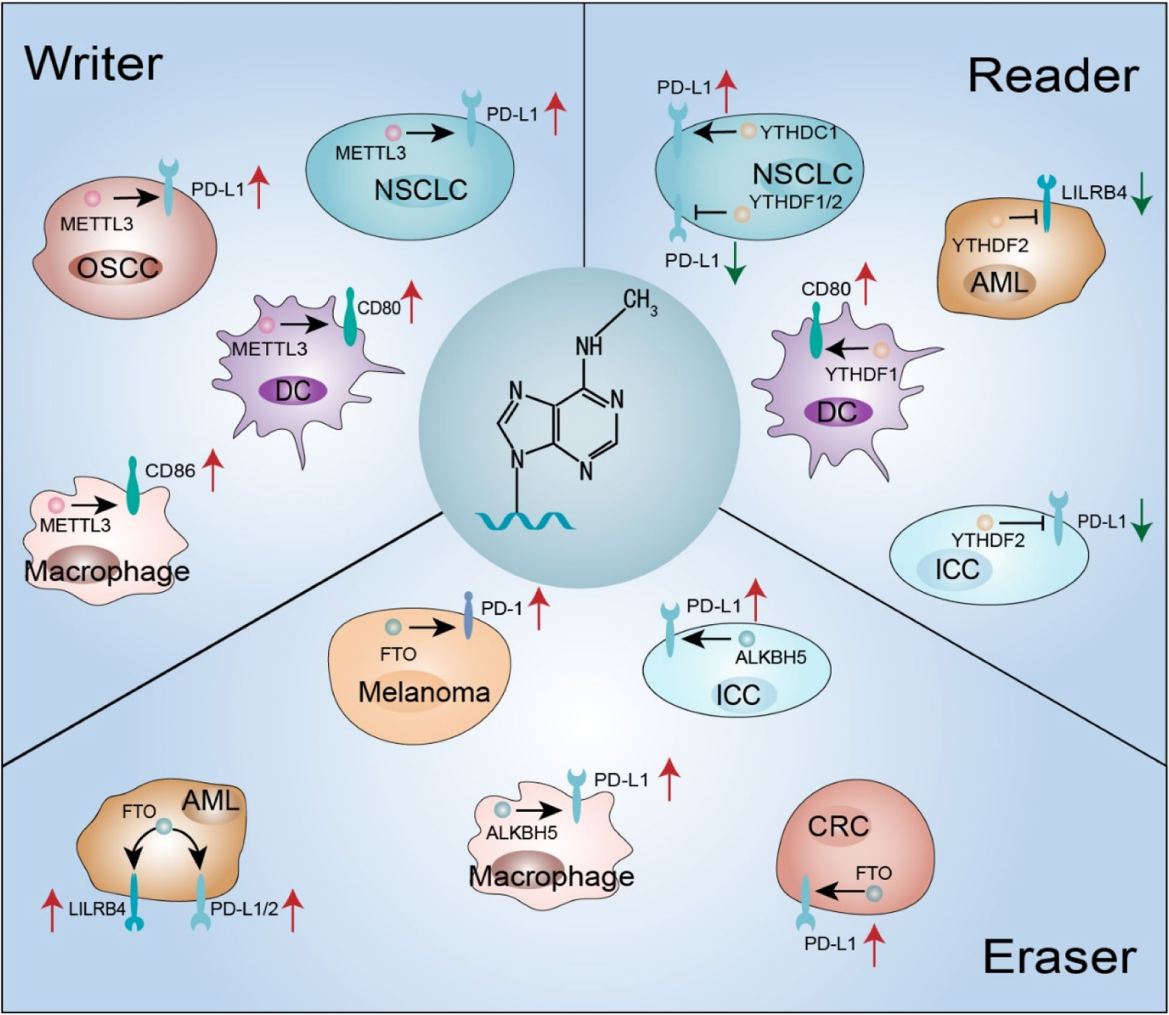
m^6^A regulators alter immune checkpoint expression in tumors. Among writers, METTL3 enhances PD-L1 expression in non-small cell lung cancer (NSCLC) and oral squamous cell carcinoma (OSCC), and augments CD80 and CD86 in DCs and macrophage, respectively. For erasers, FTO increases PD-1 and PD-L1 expression in melanoma and intrahepatic cholangiocarcinoma (ICC), respectively; FTO also ascends the expression of PD-L1, PD-L2 and LILRB4 in acute myeloid leukemia (AML). For readers, YTHDC1 escalates the expression of PD-L1 in NSCLC; YTHDF1 boosts the expression of CD80 in DCs, and descends the expression of PD-L1 in NSCLC; YTHDF2 decreases the expression of PD-L1 in ICC and NSCLC, as well as the expression of LILRB4 in AML

Compelling studies have revealed critical roles for m^6^A modification in innate and adaptive immunity, especially in modulating immune checkpoints. In this review, we elaborate on the roles of m^6^A modification in the regulation of immune checkpoints, and attempt to explore the underlying mechanisms (Table 3, Fig. 3).

**Table 3:** Functions and mechanisms of m^6^A regulators in the regulation of immune checkpoints

m^6^A	Protein	Function	Tumor/cell	Mechanism	References
Writer	METTL3	Induces resistance to anti-PD-1 therapy	CRC	Decreases IFN‐γ‐STAT1‐Irf1 signaling	[98]
	METTL3	Promotes PD-L1 expression	NSCLC	m^6^A-modified circIGF2BP3 upregulates PKP3 expression, then PKP3 engages with FXR1 to stabilize OTUB1 mRNA, thus enhancing PD-L1 expression	[90]
	METTL3	Promotes PD-L1 expression	OSCC	NM	[86]
	METTL3	Promotes CD80 expression	DCs	TLR4/NF-κB signaling	[92]
	METTL3	Promote CD86 expression	Macrophage	Stabilizes and enhances STAT1 mRNA, then stimulates IFNγ-induced CD86 expression	[95]
	METTL14	Induces resistance to anti-PD-1 therapy	CRC	Decreases IFN‐γ‐STAT1‐Irf1 signaling	[98]
Eraser	FTO	Promotes PD-1 expression	Melanoma	Via the autophagy/NF-κB/FTO axis	[32]
	FTO	Promotes PD-L1 expression	Colon cancer	IFN-γ signaling-independent manner	[103]
	FTO	Promotes PD-L1, PD-L2, LILRB4 expression	AML	Directly upregulates LILRB4 expression via an m^6^A-dependent mechanism; NM	[101]
	ALKBH5	Induces resistance to anti-PD-1 therapy	Melanoma	Modulates Mct4/Slc16a3 gene and lactate content of the TME and the composition of tumor-infiltrating Treg and MDSCs	[105]
	ALKBH5	Induces resistance to anti-PD-1 therapy	CRC	Modulates Mct4/Slc16a3 gene and lactate content of the TME and the composition of tumor-infiltrating Treg and MDSCs	[105]
	ALKBH5	Promotes PD-L1 expression	ICC	Regulates PD-L1 mRNA stability, exerts overwhelming effects on nuclear/cytoplasmic PD-L1 mRNA pool	[106]
	ALKBH5	Promotes PD-L1 expression	Macrophage	NM	[106]
Reader	YTHDC1	Promotes PD-L1 expression	NSCLC	m^6^A-modified circIGF2BP3 upregulates PKP3 expression, then PKP3 engages with FXR1 to stabilize OTUB1 mRNA, thus enhancing PD-L1 expression	[90]
	YTHDF1	Promotes CD80 expression	DCs	TLR4/NF-κB signaling	[92]
	YTHDF1	Inhibits PD-L1 expression	NSCLC	NM	[108]
	YTDHF2	Induces resistance to anti-PD-1 therapy	CRC	Decreases IFN‐γ‐Stat1‐Irf1 signaling	[98]
	YTHDF2	Inhibits PD-1 expression	melanoma	Via the autophagy/NF-κB/FTO axis	[32]
	YTHDF2	Inhibits LILRB4 expression	AML	Deteriorates LILRB4 mRNA	[101]
	YTHDF2	Inhibits PD-L1 expression	ICC	Regulates PD-L1 mRNA stability, exerts overwhelming effects on nuclear/cytoplasmic PD-L1 mRNA pool	[106]
	YTHDF2	Inhibits PD-L1 expression	NSCLC	NM	[108]

*CRC* Colorectal cancer, *NSCLC* Non-small cell lung cancer, *PKP3* Plakophilin 3, *OSCC* Oral squamous cell carcinoma, *DCs* Dendritic cells, *HCC* Hepatocellular carcinoma, *STAT1* Signal transducer and activator of transcription 1, *NK cell* Natural killer cells, *AML* Acute myeloid leukemia, *LILRB4* Leukocyte immunoglobulin-like receptor subfamily B4, *TME* Tumor microenvironment, *Tregs* Regulatory T cells, *ICC* Intrahepatic cholangiocarcinoma, *TILs* Tumor-infiltrating lymphocytes, and *NM* Not mentioned

### m^6^A writers and immune checkpoints

In recent decades, m^6^A writers, especially METTL3, have been shown to be abnormally expressed and to play critical roles in the development of a wide range of neoplasms. Dysregulated METTL3 expression in tumors exerts a great impact on antitumor immunity. In testicular germ cell tumors (TGCTs), the expression of METTL3 is decreased, which is positively correlated with the tumor infiltration of CD8 + T cells, CD4 + T cells and NK cells [83]. In cervical cancer (CC), the expression of METTL3 is upregulated, and shows a positive relation to the CD33 + myeloid derived suppressor cells (MDSCs), which function as negative regulators in antitumor immunity [84]. In BC, the expression of METTL3 is positively associated with the tumor infiltration of CD8 + T cells, helper T cells and activated NK cells, and is negatively associated with the expression of PD-L1 and PD-L2 [85]. METTL3-induced m^6^A modification, with high abundance in oral squamous cell carcinoma (OSCC), augments PD-L1 expression and restrains CD8 + T cells activation, promoting cancer cell metastasis and proliferation. Meanwhile, METTL3 knockdown results in the opposite result, suggesting METTL3 as a promising therapeutic target for OSCC [86]. Plakophilin 3 (PKP3) is a member of the arm repeat family of catenin proteins [87] and its activity is highly correlated with the progression and metastasis of various tumors. However, its role in the regulation of immune checkpoints mediated by m^6^A modification remains unrevealed [88]. A recent study reported that METTL3-methylated m^6^A modification restrains CD8 + T cell infiltration by indirectly upregulating PKP3, thereby suppressing the immune response in NSCLC. Furthermore, PKP3 integrates with the RNA-binding protein FXR1 to stabilize OTUB1 mRNA [89], which in turn elevates PD-L1 expression, indicating a positive correlation between METTL3 and PD-L1 expression in vitro [90].

Costimulatory molecules, such as CD40, CD80 and CD86, play critical roles in the immune response. However, the effect of m^6^A modification in innate immunity which is exerted by manipulating the expression of costimulatory molecules requires further investigation. DCs exhibit critical antigen-presenting functions in both innate and adaptive immunity [91]. A recent study revealed that METTL3-mediated m^6^A modification promotes DC activation and function by increasing CD40, CD80 and cytokine IL-12 expression in a NF-κB signaling dependent manner. Depletion of METTL3 dampens the antigen presentation property of DCs both in vitro and in vivo. Mechanistically, by promoting the translation of CD40, CD80 and Tirap transcripts in DCs, METTL3-mediated m^6^A modification augments T cell activation and enhances cytokine production [92]. Macrophages are critical immune cells in tissue homeostasis, inflammation, and pathologies [93]. The role and mechanism of m^6^A modification in macrophage polarization remain concealed. METTL3 expression is boosted following M1 macrophage polarization, and is proclaimed to markedly increase the stability and expression of signal transducer and activator of transcription 1 (STAT1) mRNA, which is a crucial transcription factor involved in the polarization of macrophages [94]. Disruption of METTL3 activity induces a marked inhibition of both IFN-γ-induced CD86 protein and reactive oxygen species (ROS), thereby interfering with M1 macrophage polarization [95]. Additionally, a bioinformatics study showed that METTL3 is positively correlated with the expression of CD80, CD86, PD-L1, intercellular adhesion molecule (ICAM) 1 and ICAM3 in HCC. These findings indicate that METTL3 plays a critical role in immune cell infiltration and tumor microenvironment (TME) reshaping [96].

METTL14 serves as a structural support for METTL3 to exert its unique catalytic activity. Abnormal expression of METTL14 has been observed in diverse malignancies, indicating its nonnegligible role in tumor progression [97]. However, studies on the effect of METTL14 on immunotherapy are still preliminary, and more experimental data are need. METTL3/METTL14 adjusts the TME and cytotoxic CD8 + T cell infiltration in mismatch-repair-proficient or microsatellite instability-low colorectal cancer (pMMR-MSI-L CRC). Silencing METTL3/METTL14 enhances the efficacy of immune checkpoint therapy by modulating the production of cytokines and chemokines in the TME. Mediated by YTDHF2, METTL3/METTL14 knockdown stabilizes STAT1 and Irf1 mRNAs, and promotes IFN‐γ‐STAT1‐Irf1 signaling, which in turn sensitizes tumor cells to PD‐1 inhibition. Additionally, depletion of METTL3/METTL14 sensitizes tumor cells to IFN‐γ treatment [98]. The role of METTL14 in immune cell infiltration has also recently attracted considerable attention. According to a bioinformatics study, the expression of METTL14 is downregulated in BC, and exhibits a positive association with the infiltration of CD4 + T cells, CD8 + T cells, neutrophils, macrophages and dendritic cells, as well as a negative correlation with regulatory T (Treg) cell infiltration [99]. Another bioinformatics study demonstrates that METTL14 is downregulated in clear cell renal cell carcinoma (ccRCC) and is negatively correlated with the infiltration of Treg cells [100].

### ***m***^***6***^***A erasers and immune checkpoints***

Various studies have elucidated that aberrantly expressed m^6^A erasers are associated with tumor progression by intrinsically regulating immune checkpoints [101, 102]. The oncogenic function of FTO in melanoma is manifested by increased tumor growth and inhibited host responses to anti-PD-1 therapy. The study concludes that PD-1 expression is induced via the autophagy/NF-κB/FTO axis, suggesting that the FTO/PD-1 axis is a vital component of adaptive immunity. Furthermore, FTO knockdown increases YTHDF2-mediated m^6^A modification of oncogenes, including PD-1, CXCR4 and SOX10, and sensitizes melanoma cells to IFN-γ and anti-PD-1 treatment [32].

Depletion of FTO in human colon cancer cells markedly reduces the expression of both PD-L1 mRNA and protein in an IFN-γ signaling-independent manner, highlighting the positive role of FTO in PD-L1 regulation [103]. Additionally, Liu et al. reported that FTO-mediated m^6^A demethylation allowed tumor cells to escape immune surveillance by regulating glycolytic metabolism, and restrained T cell immune responses in melanoma and lung cancer. By interfering glycolytic activity in tumors, FTO knockdown elevated the antitumor efficacy of tumor-infiltrating lymphocytes (TILs) and inhibited tumor growth in vivo. The synergistic application of FTO inhibitor and ICI restrained tumor growth, and prolonged overall survival, suggesting that targeting m^6^A modification regulators is a potential strategy to elevate immune checkpoint therapy efficacy [104]. Overexpressed FTO induced by decitabine in AML increased the expression of immune checkpoints in an m^6^A-dependent manner. Moreover, FTO depletion significantly dampened the expression of PD-L1 and PD-L2, as well as a newly characterized potent immune checkpoint, leukocyte immunoglobulin-like receptor subfamily B4 (LILRB4). The study emphasizes the critical role of FTO in regulating immune checkpoints, and propounds the bright prospect of targeting FTO in antitumor therapy [101].

ALKBH5 is reported to regulate the infiltration of Treg cells and MDSCs in the TME, and to inhibit the sensitivity of immune checkpoint therapy by modulating the m^6^A abundance and RNA stability of Mct4/Slc16a3 [105]. In melanoma cells and CRC cells, ALKBH5 knockdown altered both the recruitment of the immune cell subpopulation and the transcriptome of tumor cells. An enhanced therapeutic response to PD-1 inhibitor has also been observed in genetically mutated or ALKBH5 downregulated tumor cells, highlighting the pivotal role of ALKBH5 in the regulation of immune checkpoint therapy [105]. A recent experiment revealed that ALKBH5-mediated m^6^A demethylation directly inhibited PD-L1 expression on tumor cells and macrophages and the infiltration and cytotoxicity of T cells in intrahepatic cholangiocarcinoma (ICC). Depletion of tumor-intrinsic ALKBH5 enhanced m^6^A modification on PD-L1 mRNA, and promoted its degradation in a YTHDF2-dependent manner. This ALKBH5-PD-L1 regulatory axis may provide novel insights into immune checkpoint therapy and response prediction [106].

### ***m***^***6***^***A readers and immune checkpoints***

Readers are the true executors of m^6^A modification and control the fates of target RNAs. m^6^A modification of specific oncogenic RNA promoted PD-L1 expression and inhibited CD8 + T cell infiltration in a YTHDC1-dependent manner [90]. YTHDF1-mediated m^6^A modification suppressed the function of DCs by augmenting the translation of lysosomal protease transcripts in ovalbumin (OVA)-expressing B16 melanoma. Silenced YTHDF1 in DCs restrained lysosomal proteolysis and promoted the cross-presentation of tumor antigens, leading to promoted cross-priming of CD8 + T cells and enhanced therapeutic efficacy of anti-PD-L1. Furthermore, the combination treatment of YTHDF1 knockdown and anti-PD-L1 led to a complete tumor regression that overwhelmed the monotherapy groups evaluated, indicating that YTHDF1 depletion in combination with an ICI is a novel strategy to enhance the therapeutic efficacy [107]. Additionally, a previous study focusing on the role of m^6^A modification in the activation of DCs found that YTHDF1 was positively correlated with the translation of CD80 and CD40 mRNAs. Furthermore, YTHDF1 knockdown downregulated the expressions levels of these transcripts [92]. Another study emphasized that YTHDF1 and YTHDF2 in lung cancer cells were independent favorable prognostic indicators for recurrence-free survival. YTHDF1 and YTHDF2 expression was positively correlated with TILs density and negatively associated with PD-L1 expression. YTHDF1 and YTHDF2 knockdown enhanced PD-L1 expression in tumor cells [108].

The correlation between YTHDF2 and tumor immunotherapy has attracted considerable attention. LILRB4 is a novel immune checkpoint [109]. YTHDF2 knockdown in AML stabilizes LILRB4 mRNA, suggesting an antitumor role for YTHDF2 [101]. A bioinformatics analysis of ccRCC indicated that YTHDF2 expression was positively associated with the infiltration of immune cells, including B cells, CD8 + T cells, CD4 + T cells, macrophages, neutrophils, and DCs [110]. Additionally, positive correlations between the expression of YTHDF2 and immune checkpoints, including PD-1, TIM-3 and CTLA-4, have been observed in lower-grade glioma (LGG), emphasizing a role for YTHDF2 as a novel prognostic biomarker [111]. Moreover, another bioinformatics study demonstrated a positive correlation between the expression of m^6^A regulators and immune checkpoints, including TIM-3, LAG-3, PD-1, PD-L1, CTLA-4 and indoleamine 2,3-dioxygenase 1 (IDO1) [112]. Although the totality of the bioinformatics data indicates their correlation, the specific mechanisms by which m^6^A readers in orchestrating immune checkpoints are still largely unknown, and the research is still in its infancy. Therefore, a large amount of experimental and clinical data are needed.

### The application of m^6^A modification and ICIs for cancer treatment

**Fig. 4:**
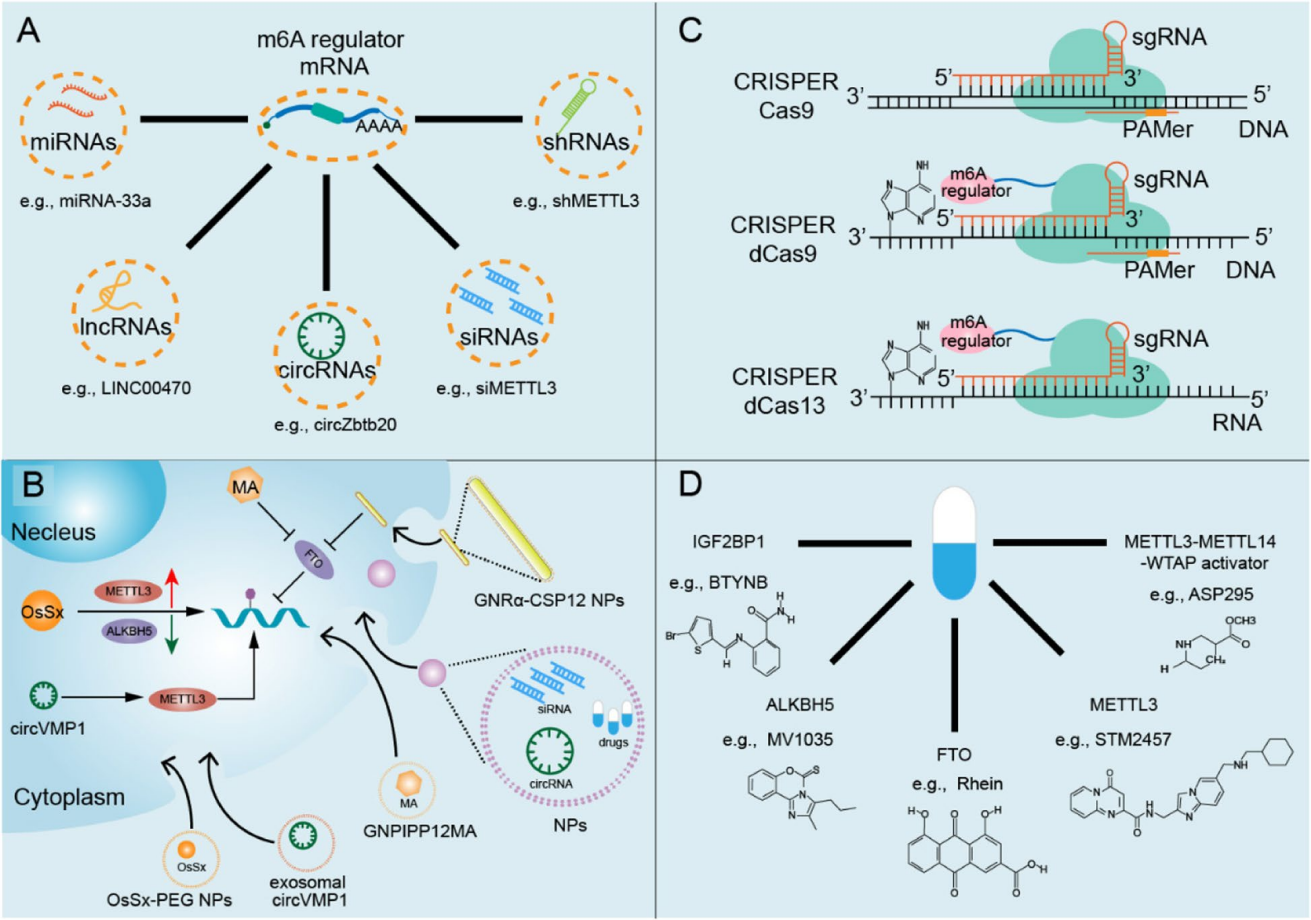
Therapeutic strategies targeting m^6^A modification. **A**. Targeting m^6^A regulators by ncRNAs, including miRNAs, lncRNAs, circRNAs, siRNAs and shRNAs to orchestrate the expression of m^6^A regulators. **B**. Nanoparticles (NPs), such as OsSx-PEG, GNRa-CSP12 and GNPIPP12MA, manipulate m^6^A modification in distinct manners; nanoparticles have also been developed to encapsulate drugs, siRNAs and circRNAs to regulate m^6^A modification. **C**. CRISPR‒Cas-based gene editing technologies for site-specific m^6^A methylation. **D**. Small-molecules targeting m^6^A regulators

Given the prominent role played by m^6^A modification in antitumor immunity, especially the manipulation of immune checkpoints, a wide range of studies have explored advantages of targeting m^6^A modification to improve the efficiency of immune checkpoint therapy (Fig. 4).

### ***Targeting m***^***6***^***A regulators by ncRNAs***

Extensive studies have elucidated the pivotal roles of m^6^A modification and its regulators in cancer development, as well as in antitumor immunity remodeling. Therefore, directly targeting m^6^A regulators may endow us with a promising strategy for cancer treatment. ncRNAs have clarified our perception of their pivotal roles in biological and pathological processes, especially their therapeutic potential in cancer treatment. In this review, we describe the currently available gene regulation strategies based on ncRNAs in preclinical studies to identify the roles of m^6^A modification in immune checkpoint therapy. MicroRNAs (miRNAs) refer to a series of noncoding RNAs that carry great gene regulation potential. They contribute to the inhibited translation or degradation of target mRNAs [113]. Notably, several miRNAs have been reported to directly target m^6^A regulators [114]. For example, a negative correlation between the expression of miR-33a and METTL3 has been reported. MiR-33a contributes to the suppression of METTL3 mRNA by directly binding to its 3' UTR, which in turn inhibits the progression of NSCLC [115]. Similarly, miR-145 has been reported to target the 3' UTR of YTHDF2, and thus dampen its expression, resulting in enhanced global m^6^A mRNA expression in HCC. Downregulated YTHDF2 is critical for inhibited tumor progression of HCC [116]. These findings of the effects of miRNAs on tumors provide valuable insights into the potential roles of miRNAs in tumor progression via fine-tuned m^6^A modification. Long noncoding RNAs (lncRNAs) are also involved in the regulation of m^6^A modification in various neoplasms. LINC00470 integrates with METTL3 and reduces the stability of the tumor suppressor phosphatase and tensin homolog (PTEN), which in turn promotes GC progression [117]. LncRNA GAS5-AS1 has been reported to associate with ALKBH5, and alter the m^6^A modification of GAS5, thus restraining cervical cancer progression [118]. CircRNAs have been reported to participate in the regulation of m^6^A modification. In a recent study of systemic lupus erythematosus (SLE), the circGARS/miR-19a signaling pathway was shown to regulate the expression of YTHDF2 to alter the activation of the immune response, which was mediated by tumor necrosis factor alpha-induced protein 3 (TNFAIP3) [119]. In group 3 innate lymphoid cells (ILC3s), circZbtb20 has been reported to promote ALKBH5-mediated demethylation on target mRNA, thus enhancing its stability [120]. RNA interferences, such as that mediated by small interfering RNA (siRNA) and short hairpin RNA (shRNA), demonstrate great therapeutic potency by orchestrating m^6^A modification. A recent study indicated that METTL3-mediated m^6^A modification promoted PD-L1 expression, inhibited CD8 + T cell infiltration, and induced tumor cell immune evasion in NSCLC. SiRNA-induced METTL3 knockdown augmented the therapeutic efficacy of anti-PD-1 therapy in an NSCLC mouse model [90]. The oncogenic role of METTL3 has also been observed in OSCC, while shRNA-mediated METTL3 knockdown dampened PD-L1 expression and intensified CD8 + T cell activation [86]. In conclusion, these findings from studies on ncRNAs have expanded our understanding of their roles in modulating epigenic regulation and provide us new therapeutic tools targeting m^6^A modification in cancer treatment.

### ***Engineered nanoparticles targeting m***^***6***^***A modification***

Nanoparticles (NPs) are promising biomaterials and drug delivery tools that effectively facilitate immunotherapy and enhance potency with reduced toxic side effects [121]. FTO inhibitor-loaded glutathione-bioimprinted nanocomposites (GNPIPP12MA) are engineered to target the FTO/m^6^A pathway with glutathione depletion for synergistically improving anti-leukemia treatment efficiency. GNPIPP12MA upregulates the global m^6^A modification level and enhances anti-PD-L1 therapy efficacy by increasing the infiltration of cytotoxic T cells [122]. Hypoxia is involved in many aspects of oncology, such as tumor proliferation, cancer stem cell maintenance, cell death, angiogenesis and drug resistance [123, 124]. Nanocatalyst named OsSx-PEG (PEG = poly(ethylene glycol)) NP as O_2_ regulator were engineered in a recent study to reverse tumor hypoxia. Mechanistically, by retaining METTL3 expression and attenuating ALKBH5 expression, OsSx-PEG NPs increase the m^6^A-dependent mRNA degradation of hypoxia-related genes, which in turn relieves the hypoxia in the TME to enhance drug efficacy and overcome resistance [125]. Another study illustrated that newly developed bovine serum albumin (BSA)-templated Au, CuS and Gd2O3 NPs alter the global m^6^A expression level, thus impairing cell proliferation, differentiation and apoptosis [126]. NPs, such as lipid-based NPs, polymer-based NPs, and inorganic NPs, can be endocytosed by tumor-associated macrophages (TAMs) and then deliver drugs or RNAs into TAMs of the TME [127–130]. For example, lipid NPs loaded with C–C motif chemokine receptor 2 (CCR2) siRNA hinder the accumulation of TAMs in the TME [127]. Polymeric NPs were developed to deliver vascular endothelial growth factor (VEGF) siRNA and placental growth factor signaling (PIGF) siRNA to both tumor cells and TAMs, leading to suppressed tumor growth and metastasis [128]. Additionally, exosomes are regarded as extracellular nanovesicles [131], and are capable of transmitting specific oncogenic RNAs to promote NSCLC progression and drug resistance by upregulating the expression of METTL3 [132]. Notably, gold nanorods loaded with chitosan and a 12-mer peptide (GNRa-CSP12) were synthesized to elevate the efficiency of targeting and internalization in AML. GNRa-CSP12 was found to impede endogenous Fe^2+^-dependent m^6^A demethylase activity, thus promoting m^6^A modification on critical targets, including PKM, CD276 and SLC2A3, which manipulated immune checkpoint pathways involved in hypoxia and glycolysis, respectively. Furthermore, GNRa-CSP12 improved the therapeutic efficacy of anti-PD-L1 by increasing CD8 + T cell and CD4 + T cell infiltration, as well as IFN-γ production [133]. The success of nanoparticles use has heralded marked progress in cancer therapy due to their great efficiency and minor side effects. However, the design of delivery technologies for m^6^A modification is still in its nascent stage. Future work needs to be devoted to the investigation of novel delivery technologies to expand and develop advanced strategies targeting the epitranscriptome.

### ***Developing m***^***6***^***A editing system***

CRISPR‒Cas9 was first described in bacterial and archaeal immunity, and is now being developed as a potent gene editing technology. Single guide RNA (sgRNA) recognizes specific DNA sequences adjacent to a specific protospacer-adjacent motif (PAM) and manipulates the DNA-cleavage activity of CRISPR‒Cas9. It is concluded that CRISPR‒Cas9 may serve as a promising approach to program m^6^A regulators in both tumor cells and immune cells. [134]. NK cells play central roles in innate immune system, and are pivotal for antitumor immunity as well [135]. It is reported that METTL3-mediated m^6^A modification promotes the antitumor immunity of NK cells in HCC. CRISPR‒Cas9 induced METTL3 knockdown impairs the homeostasis of NK cells by decreasing SHP-2 level, and inhibits their infiltration and function in the TME [136]. Depletion of METTL3/METTL14 using CRISPR/Cas9 gene editing technology sensitizes CRC cells and melanoma cells to anti-PD-1 therapy, and exhibits a synergetic effect by prolonging overall survival [98].

The application of the CRISPR‒Cas9 gene editing system has been further expanded by the construction of dCas9, which shows deficient in DNA-cleaving capacity [137]. To achieve site-specific m^6^A methylation or demethylation, a tool was engineered by tagging METTL3-METT14, FTO, or ALKBH5 to the N-terminus of RNA-targeting dCas9. These engineered m^6^A regulators can induce installation or removal of m^6^A without altering the primary sequence of target genes [138]. With the progress in gene editing technology, a new CRISPR‒Cas-based tool was developed by replacing dCas9 with dCas13 featured by smaller size, independence of PAMer oligonucleotide and minimal off-target activity [139]. Its applications and great potential have been demonstrated in previous studies in which METTL3/METTL3-METTL14 [140], FTO [50] or ALKBH5 [141] have been fused with catalytically inactive dCas13b. Ruminococcus flavefaciens Cas13d (previously named CasRx) carries the smallest dimension but most powerful targeted genome editing potency [142]. Recently, a bidirectional dCasRx-based m^6^A editing platform was engineered by conjugating dCasRx with METTL3 or ALKBH5, enabling m^6^A methylation or demethylation to occur at specific RNA sites [143]. These recently developed gene editing platforms provide optimal methods to manipulate the m^6^A modification on specific genes, offering novel strategies to elucidate the roles and underlying mechanisms of individual m^6^A regulators in fine-tuning immune checkpoints. However, experimental data are quite limited, and more research is warranted to develop novel m^6^A editors with superior precision and specificity for genome editing, especially for immune checkpoint therapy adjustment.

### ***Candidate m***^***6***^***A-targeted compounds***

A wide range of studies have confirmed that abnormally expressed m^6^A regulators contribute to carcinogenesis and progression. Therefore, compounds targeting m^6^A regulators may directly inhibit tumor cells or activate antitumor immunity in an immune checkpoint-dependent manner. To date, a series of small molecules targeting m^6^A regulators have been developed, among which FTO inhibitors are the most prolific (Table 4). Two newly discovered potent compounds, CS1 and CS2, directly bind to the enzymatic reaction center of FTO, and thus suppress the demethylase activity of FTO. In addition to dramatically inhibit the self-renewal of leukemia stem cells, CS1 and CS2 have been reported to reprogram the immune response by suppressing the expression of the recently characterized immune checkpoint LILRB4, sensitize leukemia cells to cytotoxic T cells, and abate immune escape in mice. Additionally, CS1 and CS2 display their antitumor roles in a broad range of solid tumors, including pancreatic cancer, BC and GBM [101]. Another FTO inhibitor, Dac51, restrains the glycolytic capacity of tumor cells by dampening FTO-mediated demethylation on Jun mRNA and Cebpb mRNA. It increases CD8 + T cell infiltration in the TME and acts synergistically with PD-L1 inhibitor in both melanoma cells and lung cancer cells [104]. FTO requires 2-oxoglutarate (2OG) and Fe (II) to catalyze its demethylation activity. R-2-hydroxyglutarate (R-2HG) is a 2OG analog and competitively binds to FTO, inhibiting its enzymatic activity [144]. In addition, a nonsteroidal anti-inflammatory drug called meclofenamic acid (MA) has been repurposed to inhibit the activity of FTO by specifically integrating with the active surface of FTO [31, 145]. Evidence has confirmed confirm that MA suppresses the proliferation of GBM stem cells and sphere formation in vitro and restrains the progression of GBM in vivo [146]. Moreover, two derivatives of MA, FB23 and FB23-2, exhibit significant inhibitory effects on FTO activity in AML, prostate cancer and uterine cervical cancer [147–149]. Similarly, entacapone, previously applied to Parkinson’s disease, has recently been identified as a potent FTO inhibitor [150, 151]. The combination of entacapone and imatinib (a tyrosine kinase inhibitor) is currently in an early phase I clinical study for the treatment of gastrointestinal stromal tumors [152]. Moreover, MO-I-50 has been reported to significantly suppress the survival and colony formation in triple-negative inflammatory breast cancer cells by inhibiting FTO activity [153]. Other compounds, such as Rhein, CHTB and N-CDPCB, play antitumor roles by disrupting the catalytic function of FTO [154–156].

**Table 4:** The functions of small-molecules targeting m^6^A regulators

Targeting	Compounds	Role	Functions	References
METTL3-METTL14-WTAP complex	Asp295	Activator	Promotes mRNA m^6^A modification	[159]
METTL3-METTL14-WTAP complex	Phe534	Activator	Promotes mRNA m^6^A modification	[159]
METTL3-METTL14-WTAP complex	Arg536	Activator	Promotes mRNA m^6^A modification	[159]
METTL3-METTL14-WTAP complex	Asn539	Activator	Promotes mRNA m^6^A modification	[159]
METTL3	molecule 1	Inhibitor	Reduces the abundance of mRNA targeted by METTL3 and suppresses the proliferation of leukemia cell	[146]
METTL3	molecule 2	Inhibitor	Reduces the abundance of mRNA targeted by METTL3 and suppresses the proliferation of leukemia cell	[146]
METTL3	STM2457	Inhibitor	Shows antitumor capacity against leukemia in vitro and in vivo	[160]
FTO	R-2HG	Inhibitor	Downregulates the expression of oncogene c-Myc and CEBPA, and increases the expression of antioncogene RARA and ASB2 expression; represses AML cell proliferation/viability	[144]
FTO	MO-I-50	Inhibitor	Suppresses the survival or colony forming ability of cancer cells in triple-negative inflammatory breast cancer cell lines	[153]
FTO	Rhein	Inhibitor	Competitively binds with the FTO catalytic domain and suppresses FTO function	[154]
FTO	CHTB	Inhibitor	Suppresses FTO function by binding with FTO between an antiparallel sheet and the extended C-terminal of the long loop of FTO	[155]
FTO	N-CDPCB	Inhibitor	Suppresses FTO function by binding with FTO between an antiparallel b-sheet and the L1 loop of FTO	[156]
FTO	Meclofenamic acid (MA)	Inhibitor	Specially integrates with the active surface of FTO to inhibit the activity of FTO and increase m^6^A abundance	[31, 105, 145, 147, 148]
FTO	FB23	Inhibitor	Downregulates the expression of oncogene c-Myc and CEBPA, and increases the expression of anti-oncogene RARA and ASB2 expression	[149]
FTO	FB23-2	Inhibitor	Downregulates the expression of oncogene c-Myc and CEBPA, and increases the expression of anti-oncogene RARA and ASB2 expression	[149]
FTO	CS1	Inhibitor	Directly binds to the enzymatic reaction center of FTO, interfere with its binding with target mRNA; reprograms the immune response, conquers immune escape by suppressing the expression of immune checkpoint gene LILRB4, and sensitizes leukemia cells to cytotoxic T cells	[101]
FTO	CS2	Inhibitor	Directly binds to the enzymatic reaction center of FTO, interfere its binding to target mRNA; reprograms the immune response, conquers immune escape by suppressing the expression of immune checkpoint gene LILRB4, and sensitizes leukemia cells to cytotoxic T cells	[101]
FTO	Entacapone	Inhibitor	Suppresses the function of FTO by directly binding with FTO at its cofactor and substrate binding sites	[150]
ALKBH5	ALK-04	Inhibitor	Functions synergistically with PD-1 inhibitor to inhibit melanoma	[105]
ALKBH5	MV1035	Inhibitor	Shows a favorable antitumor potency in GBM	[157]
ALKBH5	2-[(1-hydroxy-2-oxo-2- phenylethyl)sulfanyl]acetic acid	Inhibitor	Suppresses the proliferation of leukemia	[158]
ALKBH5	4-[(furan-2-yl)methyl]amino-1,2-diazinane-3,6-dione,	Inhibitor	suppresses the proliferation of leukemia	[158]
IGF2BP1	BTYNB	Inhibitor	Suppresses the proliferation of melanoma and ovarian cancer cells by downregulating the expression of targeted mRNAs	[161, 162]

*AML* Acute myeloid leukemia, *GBM* Glioblastoma, *BC* Breast cancer

Potent and selective molecules that target other m^6^A regulators are still in the stage of development, and more exploration is required. Great efforts have been directed to the identification of ALKBH5 inhibitors using high-throughput in silico screening technology. ALK-04, a small-molecule inhibitor of ALKBH5, functions synergistically with PD-1 inhibitor to suppress the growth of melanoma tumor in vivo [105]. Moreover, MV1035, another efficient ALKBH5 inhibitor, carries a favorable antitumor potency by suppressing the progression of GBM [157]. In addition, two other recently introduced ALKBH5 inhibitors, 2-[(1-hydroxy-2-oxo-2- phenylethyl)sulfanyl]acetic acid and 4-[(furan-2-yl)methyl]amino-1,2-diazinane-3,6-dione, suppressed the proliferation of leukemia with great potential [158]. Except for the small-molecular inhibitors of ALKBH5, novel agents are being investigated. As we noted above, ALKBH5 exerts its demethylase function in a 2OG and Fe^2+^ dependent manner [28]. Hence, targeting 2OG and Fe^2+^ seem to be promising strategies for targeting ALKBH5 in the future. Through in silico screening technology, four compounds, namely Asp295, Phe534, Arg536, and Asn539, are defined as derivatives of piperidine and piperazine, and act as activators of the METTL3-METTL14-WTAP complex by promoting m^6^A modification on target RNAs. However, their potential antitumor effect needs for further validation [159]. Two other METTL3 inhibitors (Compound 1 and Compound 2), which were verified in a structure-guided medicinal chemistry platform, can reduce the abundance of METTL3-methylated mRNAs and suppressing the proliferation of leukemia cells both in vivo and in vitro [146]. A recently discovered METTL3 inhibitor named STM2457 exerted a significant antitumor effect on leukemia both in vitro and in vivo, providing proof that METTL3 is a potential therapeutic target in antitumor therapy [160]. BTYNB, a potent and specific inhibitor of IGF2BP1, suppresses the proliferation of melanoma and ovarian cancer cells by downregulating the expression of target mRNAs, including c-Myc, β-TrCP1, eEF2 and E2F1 [161, 162]. Given the great antitumor effect of these agents by targeting m^6^A modification, increasing efforts have been made to explore novel m^6^A-targeted compounds, as well as their synergistic effect with immune checkpoint therapy. However, most of the current compounds are still in a preclinical stage of research, and only a few of them have been reported to be involved in the regulation of immune checkpoints. Hence, more detailed experimental data and randomized controlled clinical trials are warranted to further evaluate their roles in immune checkpoint regulation, as well as their safety and efficiency in patients.

## Conclusion and perspective

Evidence is emerging to show that m^6^A modification plays a central role in numerous regulatory pathways, and ectopic m6A modification may result in catastrophic consequences, such as carcinogenesis and cancer progression. m^6^A-based therapies are of great therapeutic potential in cancer treatment, and may sensitize tumors to immune checkpoint therapy [163]. The synergistic combination of treatments targeting m^6^A modification and ICIs apparently presents a captivating and promising prospect in cancer treatment. In this review, we summarize recent progress in understanding the function and mechanism of m^6^A modification, and further discuss its roles in orchestrating the expression and function of immune checkpoints, such as CTLA-4, PD-1 and PD-L1. Furthermore, considering the prominent role played by m^6^A modification in cancers, we suggest interventions targeting m^6^A modification as potential therapeutic strategies. Finally, numerous proofs have indicated that strategies targeting m^6^A modification may synergistically strengthen the effect of immune checkpoint therapy. These combinations may spark novel ideas for better therapeutic efficacy in clinical application.

Although strategies simultaneously targeting m^6^A modification and immune checkpoints have demonstrated tremendous potential, numerous hurdles still impede their clinical application. First of all, m^6^A regulators play dual roles in cancers. For example, METTL3 promotes cancer progression in osteosarcoma, while it plays an antitumor role in renal cell carcinoma [164, 165]. Hence, the heterogenous expression and roles of m^6^A regulators in distinct tissues or cancer types need to be taken into consideration when they are combined with ICIs in the clinical settings. In addition, the quantity of m^6^A regulators and specific agents is still limited. Thus, more efforts are urgently needed to identify novel m^6^A regulators and effective m^6^A-targeted compounds. Moreover, numerous proof-of-concept studies depict the remarkable outcomes of interventions targeting m^6^A modification in combination with ICIs. However, the efficacy and cytotoxicity of these treatments remain undisclosed. Therefore, extra experimental and clinical data are warranted for developing future tumor treatment. As concluded, studies about m^6^A modification bring a new frontier to cancer research by illustrating a comprehensive understanding of epigenetic regulation in cancer, and providing additional insight into the molecular basis of tumorigenesis and immune response. Strategies that appropriately combine m^6^A-based therapy with ICIs hold great therapeutic potential. However, the mechanisms of which m^6^A affects immune checkpoints are complicated and remain not fully understood. Numerous findings indicate that m6A modification functions in a context-dependent manner and plays dual roles. Therefore, a better understanding of the phenomenon and an improved patient selection criterion will significantly enhance the outcomes of the combined therapy that warrant further studies.

## Data Availability

Not applicable.

## Abbreviations

m^6^A: N6-methyladenosine
ICIs: Immune checkpoint inhibitors
FDA: Food and Drug Administration
irAEs: Immunotherapy-related adverse events
mRNA: Messenger RNA
ncRNAs: Noncoding RNAs
3'UTR: 3' Untranslated region
MTC: Methyltransferase complex
METTL3: Methyltransferase-like 3
METTL5: Methyltransferase-like 5
METTL14: Methyltransferase-like 14
METTL16: Methyltransferase-like 16
WTAP: Wilms tumor 1-associated protein
VIRMA: Vir-like m^6^A methyltransferase associated
ZCCHC4: Zinc finger CCHC-type containing 4
ZC3H13: Zinc finger CCCH-type containing 13
CBLL1: Cbl proto-oncogene-like 1
RBM15: RNA-binding motif protein 15
rRNAs: Ribosomal RNAs
snRNA: Small nuclear RNA
PCIF1: Phosphorylated CTD interacting factor 1
CAPAM: Cap-specific adenosine methyltransferase
tRNA: Transfer RNA
FTO: Fat mass and obesity associated protein
ALKBH5: AlkB homolog 5
hm^6^A: N6-hydroxymethyladenosine
f6A: N6-formyladenosine
m^6^Am: N6,2'-O-dimethyladenosine
GBM: Glioblastoma
BC: Breast cancer
AML: Acute myeloid leukemia
HCC: Hepatocellular carcinoma
NSCLC: Non-small-cell lung cancer
YTH: YT521-B homology
YTHDF: YT521-B homology domain family
YTHDC: YTH domain-containing proteins
IGF2BP: Insulin-like growth factor 2 mRNA-binding proteins
HNRNPs: Heterogeneous nuclear ribonucleoproteins
SRSF3: Serine- and arginine-rich splicing factor 3
carRNA: Chromosome-associated regulatory RNA
pri-miRNA: Primary miRNA
CTLA-4: Cytotoxic T lymphocyte-associated protein-4
PD-1: Programmed cell death protein-1
PD-L1: Programmed cell death ligand 1
TIM-3: T cell immunoglobulin and mucin domain-containing-3
LAG-3: Lymphocyte activation gene-3
TIGIT: T cell immunoglobulin and ITIM domain
VISTA: V-domain Ig suppressor of T cell activation
B7-H3: B7 homolog 3
BTLA: B and T cell lymphocyte attenuator
DCs: Dendritic cells
TCR: T-cell receptor
MHC: Major histocompatibility complex
APCs: Antigen-presenting cells
PP2A: Protein phosphatase 2A
SHPs: Src homology 2 domain-containing phosphatases
PI3K: Phosphatidylinositol 3-kinase
ITIM: Immunoreceptor tyrosine-based inhibitory motif
ITSM: Immunoreceptor tyrosine-based switch motif
NK cell: Natural killer cell
PLCγ: Phospholipase Cγ
TGCT: Testicular germ cell tumors
CC: Cervical cancer
MDSCs: Myeloid derived suppressor cells
OSCC: Oral Squamous Cell Carcinoma
PKP3: Plakophilin 3
STAT1: Signal transducer and activator of transcription 1
ROS: Reactive oxygen species
ICAM: Intercellular adhesion molecule
TME: Tumor microenvironment
pMMR-MSI-L CRC: Mismatch-repair-proficient or microsatellite instability-low colorectal cancer
Treg cell: Regulatory T cell
ccRCC: Clear cell Renal cell carcinoma
TILs: Tumor-infiltrating lymphocytes
LILRB4: Leukocyte immunoglobulin-like receptor subfamily B4
ICC: Intrahepatic cholangiocarcinoma
circRNA: Circular RNA
OVA: Ovalbumin
LGG: Lower-grade glioma
IDO1: Indoleamine 2,3-dioxygenase 1
siRNA: Small interfering RNA
shRNA: Short hairpin RNA
miRNA: MicroRNA
lncRNA: Long noncoding RNA
PTEN: Phosphatase and tensin homolog
NPs: Nanoparticles
BSA: Bovine serum albumin
TAMs: Tumor-associated macrophages
CCR2: C–C motif chemokine receptor 2
PIGF: Placental growth factor signaling
sgRNA: Single guide RNA
PAM: Protospacer-adjacent motif
2OG: 2-Oxoglutarat
R-2HG: R-2-hydroxyglutarate
MA: Meclofenamic acid
